# Artificial intelligence-derived left ventricular strain in echocardiography in patients treated with chemotherapy

**DOI:** 10.1007/s10554-024-03178-9

**Published:** 2024-07-23

**Authors:** Asuka Kuwahara, Yoichi Iwasaki, Masatake Kobayashi, Ryu Takagi, Satoshi Yamada, Takashi Kubo, Kazuhiro Satomi, Nobuhiro Tanaka

**Affiliations:** 1 Department of Cardiology, Tokyo Medical University Hachioji Medical Center, Tokyo, Japan; 2 Department of Cardiology, Tokyo Medical University Hospital, 6-7-1, Nishi-shinjuku, Shinjuku, Tokyo Japan

**Keywords:** Echocardiography, Global longitudinal strain, Artificial intelligence, Cancer therapy-related cardiovascular dysfunction, Heart failure

## Abstract

Global longitudinal strain (GLS) is an echocardiographic measure to detect chemotherapy-related cardiovascular dysfunction. However, its limited availability and the needed expertise may restrict its generalization. Artificial intelligence (AI)-based GLS might overcome these challenges. Our aims are to explore the agreements between AI-based GLS and conventional GLS, and to assess whether the agreements were influenced by expertise levels, cardiac remodeling and cardiovascular diseases/risks. Echocardiographic images in the apical four-chamber view of left ventricle were retrospectively analyzed based on AI-based GLS in patients treated with chemotherapy, and correlation between AI-based GLS (Caas Qardia, Pie Medical Imaging) and conventional GLS (Vivid E9/VividE95, GE Healthcare) were assessed. The agreement between unexperienced physicians (“GLS beginner”) and experienced echocardiographer were also assessed. Among 94 patients (mean age 69 ± 12 years, 73% female), mean left ventricular ejection fraction was 64 ± 6%, 14% of patients had left ventricular hypertrophy, and 21% had left atrial enlargement. Mean GLS was − 15.9 ± 3.4% and − 19.0 ± 3.7% for the AI and conventional method, respectively. There was a moderate correlation between these methods (rho = 0.74; *p* < 0.01), and bias was − 3.1% (95% limits of agreement: -8.1 to 2.0). The reproducibility between GLS beginner and an experienced echocardiographer was numerically better in the AI method than the conventional method (inter-observer agreement = 0.82 vs. 0.68). The agreements were consistent across abnormal cardiac structure and function categories (p-for-interaction > 0.10). In patients treated with chemotherapy. AI-based GLS was moderately correlated with conventional GLS and provided a numerically better reproducibility compared with conventional GLS, regardless of different levels of expertise.

## Introduction

Development of cancer treatment substantially improves prognosis of patients with cancer over the decades [1, 2]. However, some patients may face the development of cardiac therapy-related cardiac dysfunction during chemotherapy, which limits their quality of life or worsens patient prognosis [3–5]. To mitigate this anticipated risk and prevent interruption of cancer treatment, early identification of myocardial dysfunction has been recommended [6].

Global longitudinal strain (GLS) is a clinically feasible echocardiographic parameter for detecting subclinical myocardial dysfunction earlier than declines in left ventricular ejection fraction (LVEF) [7, 8]. Recent guidelines recommend using GLS for all patients before initiating cardiotoxic cancer treatment, aiming to stratify cancer therapy-related cardiovascular disease risk and identify significant changes during treatment [6]. However, GLS has not yet been fully adopted in clinical practice due to limited availability, inter-vendor variability, and the need for specialized expertise [9].

Artificial intelligence (AI) allows for learning from recorded images and performing automated image analysis for strain echocardiography [10]. Several studies have suggested that AI-based GLS reduced analysis time and the required skill, overcoming hurdles in GLS implementation [11, 12]. This method may achieve accuracy similar to manual measurements [13]. However, by semi-automatically initiating the contour before tracking, AI algorithms might reduce observer variability compared to conventional tracking [10]. Furthermore, importantly, studying the reliability of AI-based GLS versus conventional GLS holds significant clinical relevance, especially for patients undergoing chemotherapy, who often show GLS decline while maintaining preserved LVEF [7, 8].

Our aims are to investigate the correlation between AI-based GLS and conventional GLS, and whether the agreement may be influenced by cardiac structures or cardiovascular risk. Furthermore, we assess whether AI-based GLS may reduce inter-observer variability between differing expertise levels compared with conventional GLS.

## Methods

### Patient population

The present study is a retrospective cohort study including 117 patients treated with chemotherapy who underwent strain echocardiography at Tokyo Medical University Hachioji Medical Center between October 2018 and September 2020. A segment was considered missing if partly outside the image sector or if the myocardium was indistinguishable from surrounding structures due to artifacts. Those with poor image quality, defined as ≥ 3 missing segments of the 6 individual myocardial segments of the 4-chamber view, were excluded. Totally, the remaining 94 patients were included in the current analysis.

Information on medical history, laboratory results, and echocardiography were retrospectively collected. The study was approved by the local ethics committee (T2023-0031).

### Echocardiography

Echocardiographic examinations were performed by experienced examiners at the Tokyo Medical University Hachioji Medical Center using standard commercially available ultrasound equipment (Vivid E95, GE Healthcare) with a 2.5 MHz phased-array transducer and reviewed on an ECHOPAC workstation (View Pal6.14). The measurement of LVEF and left atrial (LA) volume was obtained from the modified Simpson’s rule [14].

Diastolic function was assessed from the mitral inflow pattern by pulsed-wave Doppler. Mitral annular early diastolic velocity (e′) was assessed at the septal and lateral sites of the mitral annulus using tissue Doppler imaging. E/A ratio, e′ mean and E/e′ mean ratio were calculated [15].

LV hypertrophy by indexed LV mass (LVMi) > 115 g/m² in men or > 95 g/m² in women, LA volume enlargement by indexed LA volume (LAVi) > 34 mL/m², and abnormal e′ by a septal e′ <7 cm/sec or lateral e′ <10 cm/Sects. [15, 16].

### Conventional GLS

Conventional GLS was measured using speckle-tracking analyses using the semi-automatic analysis method implemented in commercially available software (View Pal6.14). The end-diastolic (ED) was defined by the automatic ECG trigger algorithm of the analysis software. After using the ECG trigger algorithm, the timing of end-systole was manually corrected in some cases using the aortic valve closure signal obtained by pulsed-wave Doppler in the left ventricular outflow tract or by continuous-wave Doppler through the aortic valve. The observer manually corrected the region of interest (ROI) by visual assessment of the endocardial and epicardial borders. GLS was measured from the 4-chamber view, based on the maximum absolute value of strain derived from strain curves as recommended [9].

### AI-based GLS, Caas qardia [17]

**Fig. 1:**
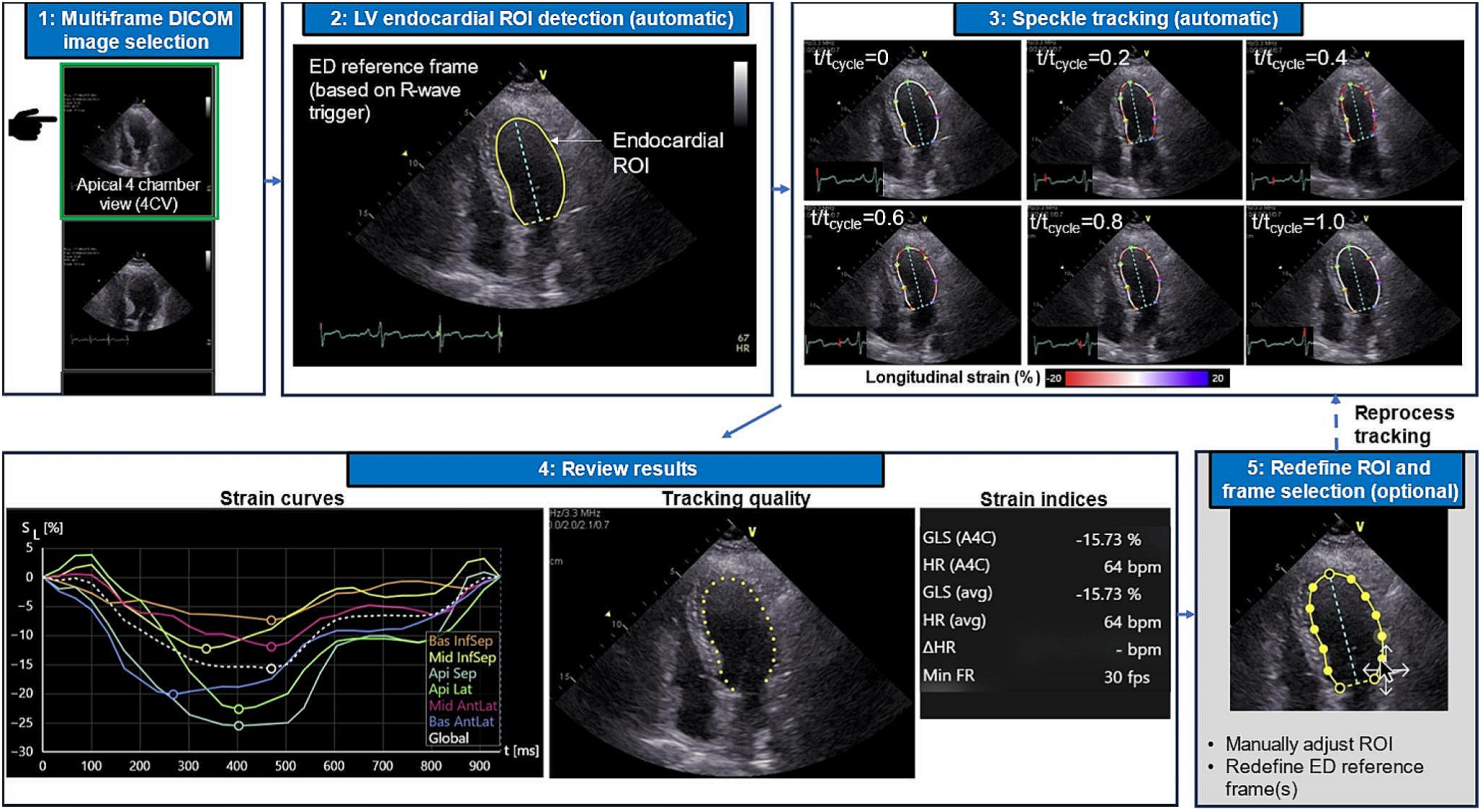
Schema of the LV longitudinal strain workflow. ROI, region of interest; t_cycle_. cardiac cycle duration; GLS, global longitudinal strain; HR, heart rate; FR, frame rate

AI-based GLS was obtained offline using a vendor-neutral speckle-tracking software platform (Caas Qardia 1.1, Pie Medical Imaging, Maastricht, NL). Core lab validation of the software platform was presented earlier [17]. In addition, the software version used in the present study contained a novel AI-based endocardial contour detection algorithm. The workflow consists of two analysis steps (Fig. 1). First, an AI algorithm automatically segmented the endocardial border at the ED reference frame using a U-Net convolutional neural network. The neural network is trained specifically for LV endocardium segmentation using standardized echocardiographic views (e.g., apical 4 chamber view). By default, the software proposes two ED frames which coincide with the first and second R-wave times of the acquired image run as determined by the ultrasound machine software. These ED frames were adjusted by the analyst if necessary. Both the ED endocardial contour and ED timings were corrected in case these were deemed to be determined incorrectly by the algorithm (Fig. 1).

The second analysis step uses the AI-based endocardial contour at the first ED phase, and the two ED phase timings as input to a speckle-tracking algorithm. By means of cross-correlation the current location of speckles is tracked for each image frame of the heart cycle at several endocardial ROI. GLS is computed based on the total myocardial line length and presented as time-strain graphs and numerical values [9].

Tracking quality was inspected visually. In case of inaccurate tracking the endocardial ROI was manually adjusted by the analyst, and tracking was reprocessed.

### Statistical analysis

Categorical variables are expressed as frequencies (percentages) and continuous variables as means ± standard deviation or median (25th and 75th percentiles) according to the distribution of the variables. The agreements between AI-based GLS and measured GLS were assessed by Bland-Altman plots with corresponding 95% limits of agreement. Inter-observer and intra-observer agreements of estimated-GLS were assessed with the intraclass correlation coefficient (ICC).

All exams for AI-based GLS were assessed by a physician (AK) with no experience with strain echocardiography (“GLS beginner”), blinded to demographic and clinical parameters. After a very short training (< 30 min of theory and practice), Inter-observer reproducibility (with an experienced cardiologist, RT) were tested with a separate complete GLS analysis of randomly selected 30 patients. A GLS beginner was considered as a physician who had never assessed GLS tracking, while an experienced echocardiographer had certification as a national echocardiographic specialist.

All analyses were performed using R version 3.4.0 (R Development Core Team, Vienna, Austria). A two-sided p-value < 0.05 was considered statistically significant. No imputation was performed.

## Results

### Baseline patient characteristics

Among patients included in the current analysis, mean age was 69 ± 12 years, 73% were female, median body mass index was 22 ± 4 kg/m², 43% had hypertension, 14% had diabetes mellitus, 52% were diagnosed with breast cancer, 77% were treated with anthracycline drug, mean systolic blood pressure (BP) was 131 ± 22 mmHg, and median b-type natriuretic peptide (BNP) was 20 (10 to 78) pg/ml. Table 1.

**Table 1:** Study Population (*N* = 94)

	Mean ± SD / *n* (%)
Age, years	69 ± 12
Female, N (%)	69 (73.4%)
Body mass index, kg/m²	22 ± 4
Body mass index ≥ 25 kg/m^2^, N (%)	23 (24.5%)
Medical history, N (%)	
Hypertension	40 (42.6%)
Diabetes	13 (13.8%)
Heart failure	2 (2.1%)
Myocardial infarction	4 (4.3%)
Cancer type	
Breast	49 (52.1%)
Lymphoma	21 (22.3%)
Gastric/colorectal	6 (6.4%)
Others*	18 (19.1%)
Anti-cancer treatments, N (%)	
Surgical intervention	49 (52.1%)
Radiotherapeutic intervention	13 (13.8%)
Chemotherapy, N (%)	
Anthracycline chemotherapy	72 (76.6%)
HER2-targeted therapy	21 (22.3%)
Other targeted therapy	36 (38.3%)
Systolic BP, mmHg	131 ± 22
Diastolic BP, mmHg	71 ± 14
Heart rate, mg/dl	74 ± 15
Laboratory data	
Creatinine, mg/dl	0.8 ± 0.5
Hemoglobin, g/dl	11.7 ± 2.0
BNP, pg/ml	20 (10 to 78)
Echocardiogram	
LV end-diastolic diameter, mm	44 ± 5
LV end-systolic diameter, mm	29 ± 5
LV ejection fraction, %	63 ± 8
LV mass index, g/m²	83 ± 22
LV hypertrophy, N (%)	13 (13.8%)
LA volume index, ml/m²	28 ± 9
LA volume index > 34 ml/m², N (%)	21 (22.3%)
Septal e′, cm/sec	6.8 ± 2.0
Lateral e′, cm/sec	8.7 ± 2.7
Septal e′<7 cm/sec or lateral e‘<10 cm/sec, N (%)	63 (70.0%)
AI-based GLS, %	-19.0 ± 3.7
Conventional GLS, %	-15.9 ± 3.4

Values are expressed as median (25th to 75th percentile) or n (%)

HER2, human epidermal growth factor receptor-2; BP, blood pressure; BNP, B-type natriuretic peptide; LV, left ventricular; LA, left atrial; GLS, global longitudinal strain

*Others included, urologic, hepatic, lung, hematologic (i.e., multiple myeloma and myelodysplastic syndromes) cancers

For echocardiographic parameters, the mean LVEF was 63 ± 8%, 14% of patients had a LVH, 22% had LA enlargement, and 70% had increased LV stiffness. Table 1.

### Correlation between AI-based GLS and conventional GLS

**Fig. 2:**
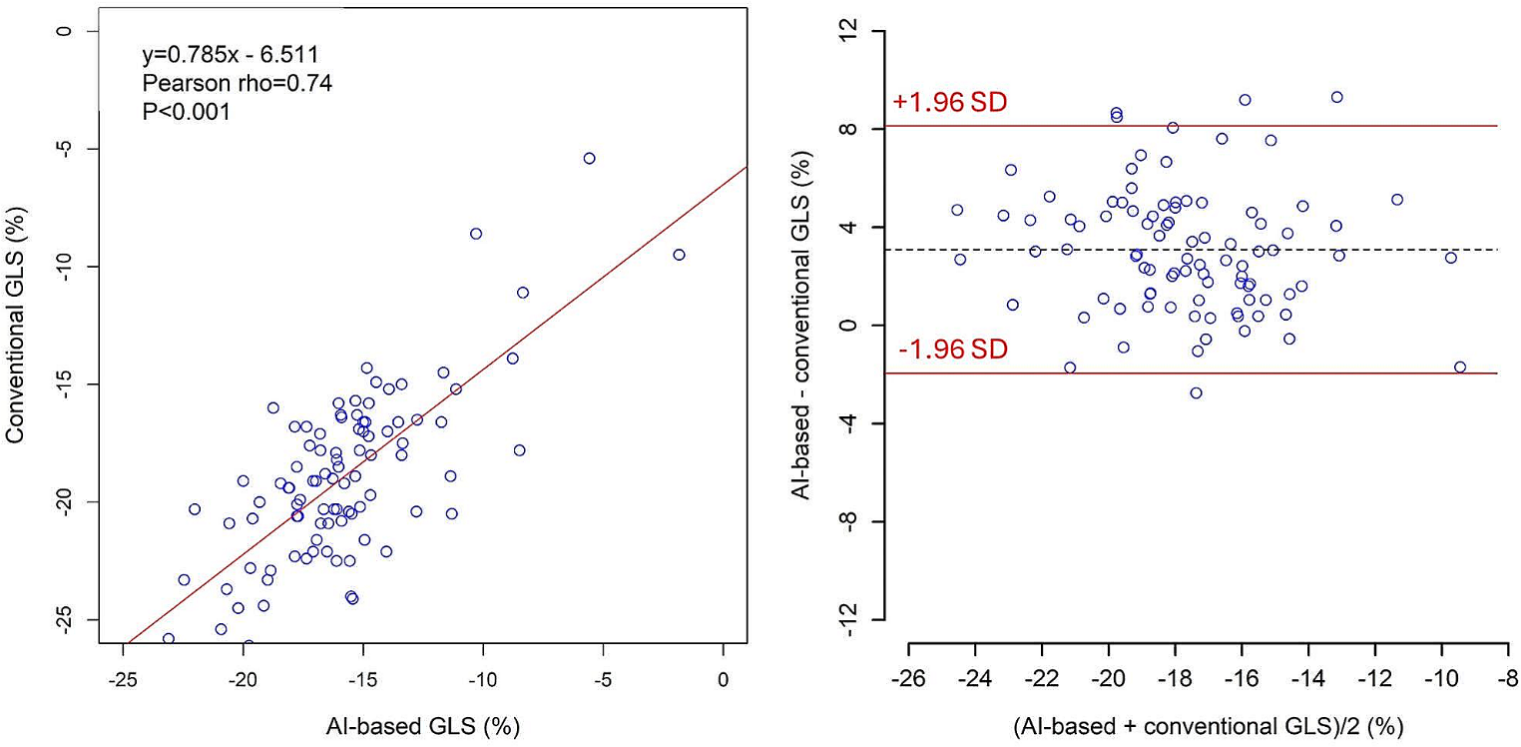
Correlation plots and Bland-Altman plots between AI-based and conventional GLS. GLS, global longitudinal strain

Mean GLS in the entire population was − 19.0 ± 3.7% and − 15.9 ± 3.4% for the AI method and the conventional method, respectively. There was a significant correlation between the methods (rho = 0.74; *p* < 0.01, Fig. 2). The Bland-Altman analysis of between method differences showed a bias of -3.1% with limits of agreement ± 5.0%. Figure 2.

### Agreements between AI-based and conventional GLS across different cardiac structure, function and cardiovascular diseases/risks

The agreement between AI-based and conventional GLS across different categories of abnormal cardiac structure and function (i.e., LV systolic function, hypertrophy, and regional wall motion abnormalities, and diastolic function) and presence/absence of cardiovascular disease and/or risk factors (i.e., hypertension, diabetes, heart failure, myocardial infarction) are presented in Table 2. Overall, the agreements between AI-based and measured GLS were not modified by these categories (p-for-interaction > 0.10). Table 2.

**Table 2:** Agreement between AI-based GLS and conventional GLS across cardiac structure, function and cardiovascular diseases/risk factors

		Number	Average diff	Limits of agreement	Pearson rho	*P*-for-interaction
LVEF	≤ 60%	30	-2.12	-7.62 to 3.39	0.74	0.74
	> 60%	64	-3.55	-8.14 to 1.04	0.62	
LV hypertrophy	Absence	81	-3.29	-8.43 to 1.86	0.67	0.40
	Presence	13	-1.89	-5.63 to 1.84	0.91	
LAVi	≤ 30 ml/m^2^	56	-3.02	-8.22 to 2.18	0.68	0.38
	> 30 ml/m^2^	38	-3.21	-8.08 to 1.67	0.80	
Septal e′<7 cm/sec or lateral e‘<10 cm/sec	Absence	27	-3.38	-8.12 to 1.36	0.76	0.32
	Presence	63	-3.08	-8.27 to 2.11	0.62	
LV asynergy	Absence	79	-3.26	-7.94 to 1.42	0.61	0.55
	Presence	15	-2.22	-8.82 to 4.38	0.72	
Cardiovascular disease and/or risk factors*	Absence	43	-2.61	-7.49 to 2.27	0.64	0.53
	Presence	51	-3.67	-8.73 to 1.39	0.79	

LVEF, left ventricular ejection fraction; LV, left ventricular; LAVi, left atrial volume index

*Cardiovascular diseases and/or risk factors included hypertension, diabetes, myocardial infarction and heart failure

### Agreements between GLS beginner and experienced echocardiographer using AI-based and conventional GLS

**Fig. 3:**
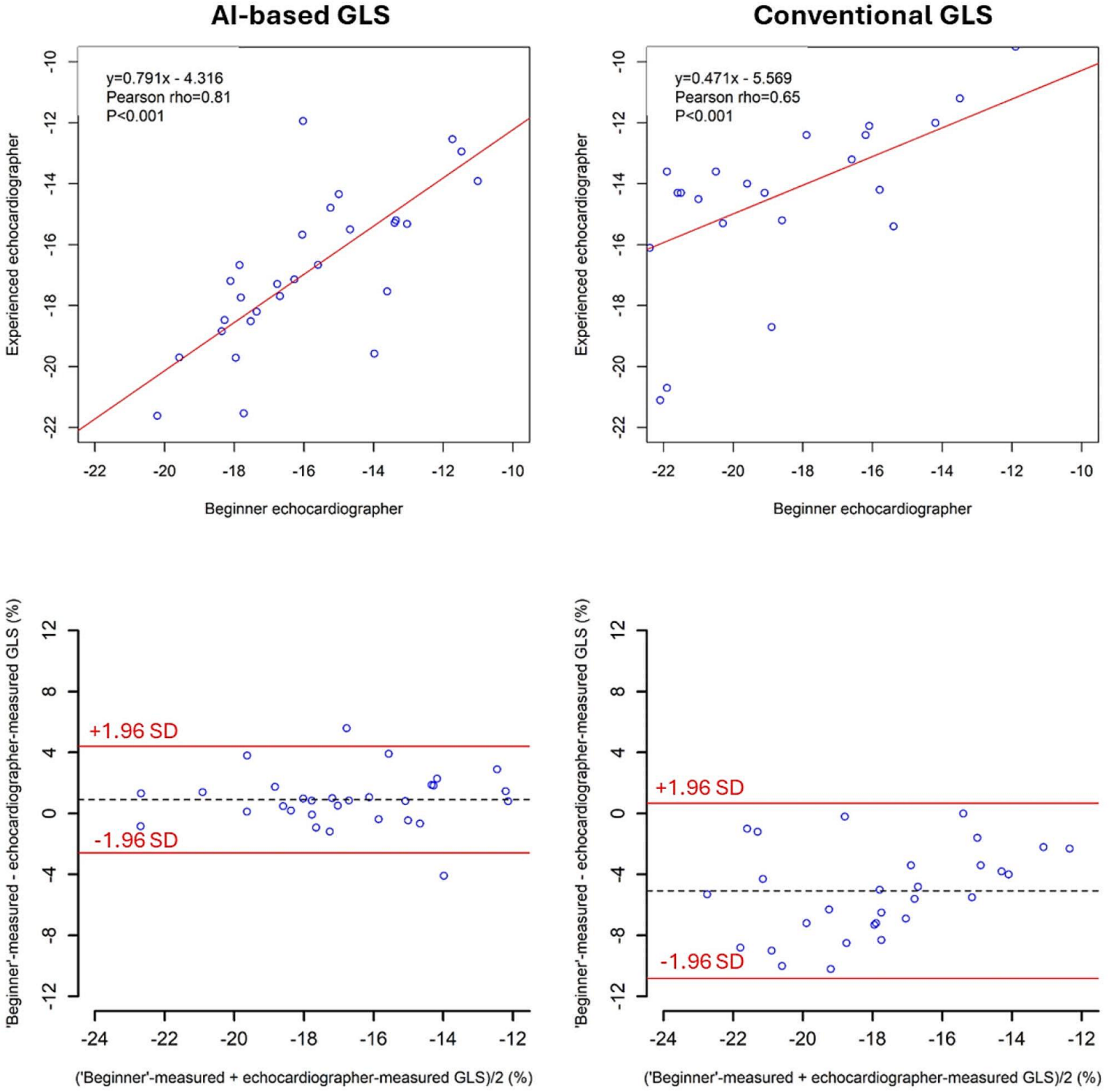
Agreements between GLS beginner and experienced echocardiographer using AI-based and conventional GLS. AI, artificial intelligence; GLS, global longitudinal strain

Inter-observer ICC coefficients between GLS beginner and experienced echocardiographer showed numerically higher reproducibility in the AI method than the conventional method (inter-observer agreement [95%CI] = 0.81 [0.64 to 0.90] in the AI method vs. 0.62 [0.34 to 0.80] in the conventional method). The Bland-Altman analysis of between examiner differences showed a bias of 0.91% with limits of agreement ± 3.5% in AI method, and a bias of -5.07% with limits of agreement ± 5.74% in conventional method (Fig. 3).

## Discussion

In the present study, our results showed that, in patients eligible for GLS-based assessments, AI-based GLS was moderately correlated with conventional GLS. The agreements were consistent across abnormalities of cardiac structure and function (i.e., LV systolic function, LV hypertrophy, LA enlargement and diastolic dysfunction), and cardiovascular diseases/risks. Additionally, the agreement between GLS beginner and an experienced echocardiographer was numerically better in the AI-based method than the conventional method. These findings support the utility of AI-based GLS and enhance its application among patients undergoing for chemotherapies in clinical practice.

The incorporation of AI into cardiovascular image analysis software is ongoing [18]. Its fundamental applications include the assessment of cardiac function by LVEF and GLS [10, 18]. There are roughly two AI approaches for echocardiography-based GLS calculation. The first approach, such as used in the present study, utilizes deep learning to perform endocardial or full cardiac wall segmentation. This segmentation subsequently serves as input for a motion calculation algorithm, (e.g., by speckle tracking or optical flow methods), estimating the displacement and translation of the cardiac wall throughout the cardiac cycle [19, 20]. The second approach utilizes deep learning for both the segmentation and motion tracking steps [21].

To date, performance and validation data of these methods often comprise of head-to-head comparison between the AI-based GLS method and conventional-based method. Herein, correlation coefficients between the methods ranged from moderate to excellent ranging between 0.54 and 0.93, respectively [19–22]. For example, in a community-based cohort including 561 patients with a risk of developing heart failure, the agreement between manual and fully automated GLS showed fairness (rho = 0.69), and its agreement was improved with semi-automated GLS (rho = 0.84). However, the limited expertise in performing strain echocardiography may constrain the generalizability of these findings [22]. With this regard, our results showed numerically better reproducibility of AI-based GLS method than conventional GLS method. These findings may be explained by the fact that our AI approach can result in a hybrid approach combining AI for segmentation as initialization step for subsequent strain calculation, subsequently reducing the inter observer variability, and suggested this AI tool could be more user-friendly, particularly for unexperienced physicians.

Patients with abnormalities of LV structure and function were more likely to have lower value of GLS, compared with those not [23, 24]. Along with technical issues (i.e., tracing process, LV segment identification), different cardiac morphology and/or cardiovascular diseases may impact the agreement between AI-based GLS and conventional GLS [20]. Nonetheless, our results showed that the agreements between two GLS approaches were consistent across different abnormal cardiac structure, function and cardiovascular diseases/risks. These results are reassuring and may boost the applicability of our AI-based GLS approach.

Previously, the EACVI/ASE Strain Standardization Taskforce assessed the inter-vendor variability of nine conventional-based GLS method [25]. Future investigations by the Taskforce could reveal the performance and robustness characteristics of the different AI approaches for calculating GLS, i.e., based fully on deep learning, or a hybrid approach combining AI for segmentation as initialization step, and subsequent using the widely adopted speckle tracking or optical flow methods. Importantly, the merits of our AI-based GLS lie in assessing GLS values within pre-existing images through web-based software (eliminating the need for desktop installation) and in achieving better reproducibility of AI-based GLS across both inexperienced and experienced echocardiographers, compared to conventional GLS. Patients who are candidates for cancer treatment are recommended to undergo GLS measurements at least every three months during the treatment period [26, 27]. Therefore, our AI-based echocardiogram may hold potential for increasing the relevance of GLS assessments for patients treated with chemotherapies in clinical practice.

### Limitations

The results should be interpreted in light of the following limitations. This retrospective study has a small sample size, which may limit the generalizability of our findings and necessitate further statistical power to confirm them. AI-based GLS value was determined through assessment with good image quality, < 2 missing segments of the 6 individual myocardial segments in the 4-chamber view. All echocardiographic images were captured by experienced echocardiographic examiners; hence, any differences of generating the images due to varying levels of expertise were not accounted for in this analysis. Nevertheless, the utilization of an AI-based methodology might offer a solution to the challenge of precisely delineating the endocardium. The timing of performing echocardiography, particularly the duration after chemotherapy may impact patient cardiac function, however, we lacked a consistent duration period between chemotherapies and echocardiography assessment. Additionally, the longer training period for GLS beginners may influence our results. An adequately powered prospective study following structured training should be warranted.

Although GLS value may vary with vendors and examiners [25, 28], in the current analysis, GLS values, which experienced examiners measured using the conventional method, were considered as a standardized value. The influence of observer experience level on performance and the concordance of AI-based GLS values with those from different imaging modalities, such as cardiac magnetic resonance imaging, are sparsely reported and warrant further investigation [29].

## Conclusions

In patients treated with chemotherapy. AI-based GLS was moderately correlated with conventional GLS. The agreement of AI-based GLS values measured by beginner and experienced echocardiographers was numerically better than that of conventional GLS.

## Data Availability

No datasets were generated or analysed during the current study.
